# Vidi, vini, vinci: External ophthalmomyiasis infection that occurred, and was diagnosed and treated in a single day: A rare case report

**DOI:** 10.4103/0974-620X.57313

**Published:** 2009

**Authors:** Kamlesh Thakur, Gagandeep Singh, Smriti Chauhan, Anuradha Sood

**Affiliations:** Department of Microbiology, DRRPGMC, Tanda, India; 1 I.G.M.C., Shimla, Himachal Pradesh, India

**Keywords:** *Acute presentation*, *maggots*, *Oestrus ovis*, *ophthalmomyiasis*

## Abstract

Ophthalmomyiasis is an infestation of eye with larvae or maggots of certain flies. Oestrus ovis (sheep nasal botfly) belonging to family Oestridae is the most common cause of human myiasis. We describe here an acute presentation of a case of external ophthalmomyiasis, i.e., infestation of conjunctiva due to first instar larvae of Oestrus ovis. In this case report the occurrence, diagnosis and treatment all took place in the setting of a single day. Prompt treatment by removal of larvae mechanically followed by instillation of antibiotic and steroid eye drops helped to prevent serious complications. The taxonomic identification of fly is also important as some fly species are capable of penetrating deeper tissues of eyes, which is sight threatening.

## Introduction

Myiasis is the infestation of humans and vertebrate animals with dipterous larvae (maggots).[1] In humans, invasion of skin is the most common, though larvae have been recovered from many organs i.e. eyes, ears, nose, intestines and urogenital tract.[2] Ophthalmic involvement is classified as external, internal or orbital ophthalmomyiasis, based on the site of larval invasion. *Oestrus ovis* (Class: Insecta, Order: Diptera, Family: Oestridae) also known as the sheep nasal botfly, is one of the most common causes of ophthalmomyiasis.[3] It is an obligate parasite of sheep and goats. Occasionally, man acts as an accidental host. Ocular myiasis is more frequent in tropical than temperate regions.[4,5] The clinical presentation is usually similar to that of a viral or allergic conjunctivitis with foreign body sensation, irritation, redness, photophobia and may be mistaken for periorbital cellulitis.

Only scanty reports of ophthalmomyiasis have been documented in literature all over the world.[4‐6] Here we report a case of external ophthalmomyiasis (conjunctival) caused by *O. ovis*.

## Case Report

A 17-year-old school girl presented to the Ophthalmology outpatient department with a history of foreign body sensation, irritation and watery discharge in right eye. She developed these symptoms after exposure to a dust storm while she was playing in a school campus. She denied being struck in the eye by an insect at that time. The patient was brought to the hospital within one hour of the incident. There was no history of recent travel or exposure to farm animals like sheep etc. On ophthalmological examination, unaided visual acuity was 6/5 bilaterally. The conjunctiva was slightly congested along with small, transparent, moving maggots in the fornix, on the plica interna and under the upper eyelid. The organism moved away from the light beam of the slit lamp. The cornea, anterior chamber and fundus examination were normal.

**Figure 1:**
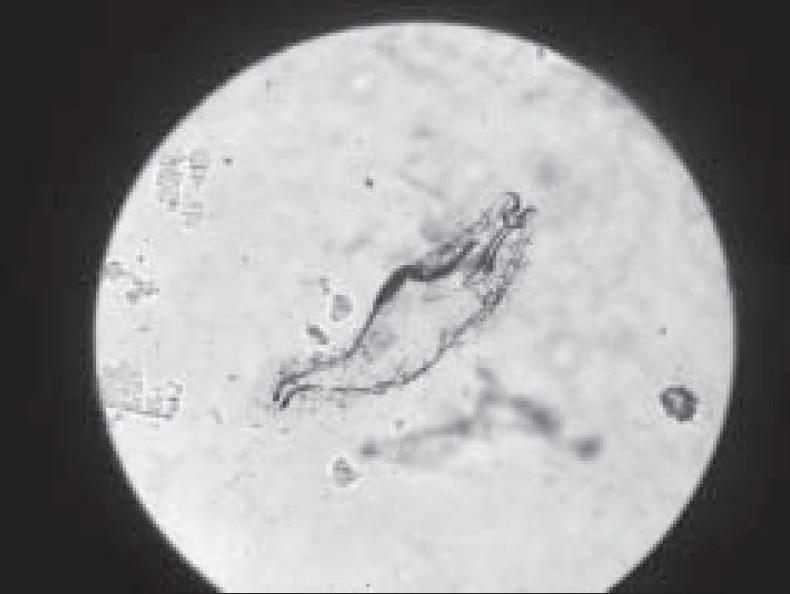
Maggot removed from the patient showing translucent, segmented body and two large dark oral hooks connected to a white cephalopharyngeal skeleton

**Figure 2:**
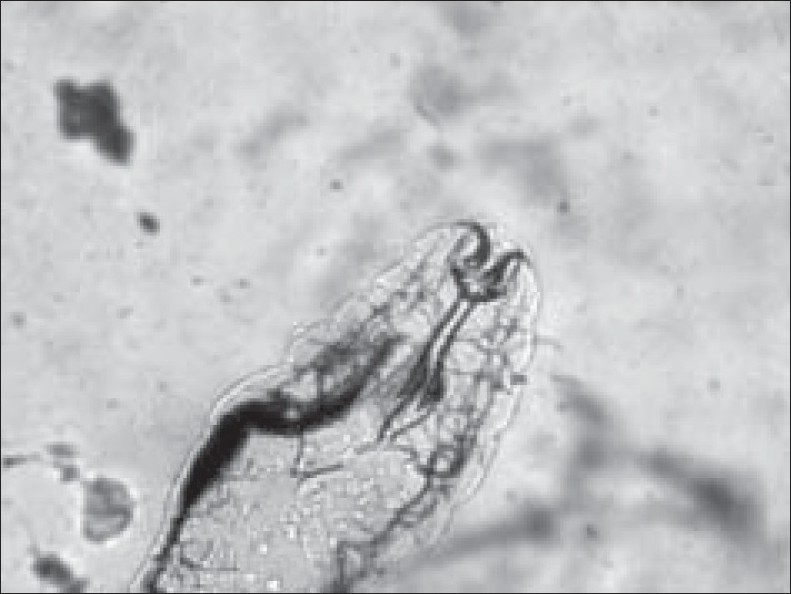
Magnified view showing anterior part of the maggot with a pair of dark oral hooks

The retrieved larvae were sent to the microbiology laboratory in the normal saline for identification. These were seen as white, about 1.5 mm × 0.5 mm in size, motile larvae in wet mount. Later on these were mounted on slides and presumptively identified microscopically as larvae of *O. ovis*, and subsequently confirmed by an entomologist at the local university as first instar larvae of *O. ovis*. The prominent identifying features of the larvae included the segmented translucent body, and white cephalopharyngeal skeleton with characteristic pair of curved, dark oral hooklets [Figures 1 and 2].

About 15 larvae were removed with the help of forceps from the right eye after instillation of topical anesthetic (Xylocaine 4%). The eye was irrigated with 500 mL of normal saline. The patient was advised topical ciprofloxacin 0.3% and dexamethasone 0.1% four times a day to prevent secondary bacterial infection and to reduce inflammation. On follow-up examination the following day, no residual larvae could be found and ocular symptoms had largely resolved.

## Discussion

Myiasis is an infestation of live human or vertebrate animals with dipterous fly larvae, which for certain period of their life, feed on dead or living tissues or ingested food of their hosts.[2] Myiasis in humans is clinically categorized in six ways: dermal and subdermal, facial cavity, wound or traumatic, gastrointestinal, vaginal and generalized myiasis. Human myiasis is caused by three dipteran families. These families include Oestridae, Calliphoridae and Sarcophagidae. List of important species of flies causing ophthalmomyiasis along with their common names is shown in Table 1.

**Table 1:** List of important species of flies causing ophthalmomyiasis

*Family*	*Scientific name*	*Common name*
Oestridae	Oestrus ovis	Sheep bot fly
	Dermatobia hominis	Human bot fly
	Cuterebra emasculator	Squirrel bot fly
	Gasterophilus intestinalis	Horse bot fly
	Hypoderma lineatum	Common cattle grub
Calliphoridae	Lucilia cuprina	Australian blowfly
	Chrysomya bezziana	Old world Screwworm
	Cochliomyia hominivorax	Primary Screwworm
Sarcophagidae	Sarcophaga aurifrons	Grey-Striped Fly

The frequency of ophthalmomyiasis is greater in those regions where there are high ratios of sheep to people. Most affected patients are involved in agricultural activities and/or sheep raising. It is thought that in these situations people have closer contact with sheep and goats[7] but this is not a necessary precondition[2] as in our patient. Other contributory factors to human infestation may be poor hygienic conditions and debilitating diseases such as chronic infections, malignancies and HIV infection. The gravid adult female fly swarms around the head of animals and ejects the first instar larvae onto nostrils of the animals as milky fluid. In man, the larvae cannot survive beyond the first stage and are believed to die within 10 days.[1] Therefore, the infestation is of short duration.

Severity of myiasis depends on the location of the infestation.[4‐6,8] The ophthalmic sequelae are mostly benign and self limiting. However in internal ophthalmomyiasis, caused by larvae from some other species such as *Hypoderma* (or cattle grub), the larvae penetrate the sclera and burrow in the subretinal space. This can lead to iridocyclitis, endophthalmitis or loss of vision. In orbital ophthalmomyiasis, larvae invade orbital contents.

The symptoms of external ocular myiasis include acute ocular foreign body sensation, irritation, redness, lacrimation, photophobia and reduced visual acuity. Signs include eyelid edema with erythema, conjunctival edema, hemorrhages, chemosis and superficial punctuate keratitis. These clinical features may be mistaken for a periorbital cellulitis.[9] The treatment of external ophthalmomyiasis includes mechanical removal of larvae. The use of topical anesthetics or an anticholinesterase agent or both which paralyze the larvae has been recommended to facilitate their removal. Liquid paraffin has also been used. It cuts off the oxygen supply thereby killing the larvae. It is prudent to remove the larvae from conjunctiva promptly. Topical steroids and antibiotics relieve symptoms and prevent secondary bacterial infection respectively. Topical ivermectin has been shown to be effective in treating myiasis.[10] Follow-up examination is recommended to avoid the possible complication of internal ophthalmomyiasis.
